# Heart Rate Variability Is Associated with Survival in Patients with Brain Metastasis: A Preliminary Report

**DOI:** 10.1155/2013/503421

**Published:** 2013-09-12

**Authors:** Yu-Ming Wang, Hau-Tieng Wu, Eng-Yen Huang, Yu Ru Kou, Shu-Shya Hseu

**Affiliations:** ^1^Institute of Physiology, National Yang-Ming University, Beitou, Taipei 11221, Taiwan; ^2^Department of Radiation Oncology, Kaohsiung Chang Gung Memorial Hospital and Chang Gung University College of Medicine, Niao-Sung, Kaohsiung 83306, Taiwan; ^3^Department of Mathematics, Stanford University, Stanford, CA 94305, USA; ^4^School of Traditional Chinese Medicine, College of Medicine, Chang Gung University, Kwei-Shan, Taoyuan 33302, Taiwan; ^5^Department of Anesthesiology, Taipei Veterans General Hospital, Beitou, Taipei 11217, Taiwan

## Abstract

Impaired heart rate variability (HRV) has been demonstrated as a negative survival prognosticator in various diseases. We conducted this prospective study to evaluate how HRV affects brain metastasis (BM) patients. Fifty-one BM patients who had not undergone previous brain operation or radiotherapy (RT) were recruited from January 2010 to July 2012, and 40 patients were included in the final analysis. A 5-minute electrocardiogram was obtained before whole brain radiotherapy. Time domain indices of HRV were compared with other clinical factors on overall survival (OS). In the univariate analysis, Karnofsky performance status (KPS) <70 (*P* = 0.002) and standard deviation of the normal-to-normal interval (SDNN) <10 ms (*P* = 0.004) significantly predict poor survival. The multivariate analysis revealed that KPS <70 and SDNN <10 ms were independent negative prognosticators for survival in BM patients with hazard ratios of 2.657 and 2.204, respectively. In conclusion, HRV is associated with survival and may be a novel prognostic factor for BM patients.

## 1. Introduction

Brain metastasis (BM) is the most common intracranial malignancy, developing in 20%–40% of all cancer patients during the course of the disease. Typical BM treatments include surgical resection, stereotactic radiosurgery, and whole brain radiotherapy (WBRT) [1]. Medical professionals should choose a treatment according to the survival prognosis of the patients [2]. A widely used prognostic index, referred to as the radiation therapy oncology group recursive partitioning analysis (RPA) classification, was published by Gasper et al. [3] in 1997. Based on the RPA, patients are classified into 3 categories: RPA I includes patients who are <65 years old, with a Karnofsky performance status (KPS) ≥70, a controlled primary, and no extracranial metastasis; RPA III includes patients who demonstrate a KPS <70; all remaining patients are classified as RPA II [3]. Since 1997, several prognostic indices have been proposed, assessing the number of metastatic brain lesions, the largest intracranial lesion size, and the systemic disease status [4–6]. Although these prognostic indices are widely used in clinical settings, substantial proportion of erroneous survival prediction exists, for example, those reported by Nieder and Molls and Villà et al. [7, 8]. Erroneous prediction can eventually lead to an inadequate choice of treatment [7, 9]. Therefore, it is worthwhile to seek a novel parameter that accurately predicts survival in BM patients.

Heart rate variability (HRV) is a well-known physiological phenomenon in which the time interval between heart beats varies; in other words, the normal-to-normal beat (NN) interval sporadically varies. The HRV reflects the complexity of the physiological system that controls homeostasis in the human body. Numerous methods have been proposed to quantify and analyze the HRV, and time domain measures are widely applied. By using the standardized time domain analysis [10], several HRV indices can be generated from the electrocardiogram (ECG), such as the standard deviation of the NN interval (SDNN), root mean square standard deviation of the NN interval (RMSSD), triangular interpolation of the NN interval histogram (TINN), number of pairs of adjacent normal-to-normal intervals differing by more than 50 ms (NN50), and the proportion of NN50 divided by total normal-to-normal intervals (pNN50) [10].

Previous studies have demonstrated that HRV provides a prognosis for various diseases including myocardial infarction, diabetes mellitus, and infection and can be used to attain the prognostic outcome in intensive care units [11–14]. Recently, scholars have examined how HRV affects cancer patients. Couck and Gidron and Kim et al. demonstrated that cancer patients possess a relatively lower HRV compared with that of healthy people [15, 16]. Among cancer patients, advanced stage patients possess a lower HRV than do those in the early stages of cancer [15]. In addition, Mouton et al. showed that low level of HRV can predict subsequent cancer progression by examining the increasing tumor markers [17]. Furthermore, impaired HRV has been correlated with a short survival time and is a poor prognosticator in patients with advanced cancer [16, 18, 19]. Although these studies have established the relationship between HRV and cancer survival, the effects of HRV on BM patients remain uninvestigated.

Based on the need for a novel prognosticator of BM patients survival, and the significant relationship between HRV and cancer survival, we conducted this prospective study to evaluate the hypothesis that time domain HRV indices are associated with survival and can be used as a prognostic factor in BM patients.

## 2. Materials and Methods

### 2.1. Patients

This prospective study was conducted between January 2010 and July 2012. Patients who were diagnosed with metastatic brain cancer and referred for palliative WBRT were enrolled in the study. To minimize the potential influence of medical treatments on HRV, patients who took antihypertensive drugs, sedatives, or antiarrhythmic drugs were excluded from the study. Patients who had undergone previous central nervous system or chest operation or radiotherapy (RT) were also excluded. For the limitation of HRV processing, patients with arrhythmia or too many premature ventricular contractions (defined as >1% of all beats) in the ECG recording were included in the study but excluded from the following analysis. The brain metastases were confirmed using contrast-enhanced magnetic resonance images or computed tomography (CT) scans. All patients underwent complete physical examination and ECG examination before WBRT was initiated. All clinical factors, previous local/systemic cancer treatment histories, and brain images were carefully reviewed for each patient. The study protocol was approved by the Institutional Review Board of Chang Gung Memorial Hospital (98-3760B), the study design was explained to each patient, and a written informed consent was obtained from all participants.

### 2.2. ECG Acquisition and Analysis

The ECG examinations were performed before the WBRT was initiated. The ECGs were executed after the patients had rested for 5 minutes in a quiet examination room in the supine position, and after their heartbeats and respiration rhythms had stabilized. The ECG signals were acquired using a commercialized ECG recorder (MyECG E3-80 portable ECG recorder; MSI, New Taipei City, Taiwan) for 5 minutes. The digital signals were saved at 12-bit resolution and a sampling rate of 1000 Hz. Next, the R peak of each valid QRS complex was detected using MyECG E3-80 portable ECG software (MSI, New Taipei City, Taiwan) and labeled with a time stamp. The time intervals between successful adjacent R peaks were collected for the normal-to-normal R-R interval time series. The time series were subsequently calculated using the same software according to the recommendations of the Task Force of the European Society of Cardiology and the North American Society of Pacing and Electrophysiology [10]. By using the time domain HRV analysis, several parameters were generated for further analysis including the SDNN, RMSSD, TINN, NN50, and pNN50. Because no previous reference could be used to stratify the BM patients, the values of the lower one-third of SDNN and RMSSD were used as the cutoff values for prognostic stratification.

### 2.3. Radiotherapy Treatment and Follow-Up

All patients were simulated by a CT simulator with slice thickness of 3.75 mm in supine position and immobilized with a thermoplastic cast. Then, patients underwent WBRT using 6- or 15-megavoltage photon irradiated via a conventional bilateral opposed helmet field. The radiotherapy dose to the whole brain was 30 Gy to 37.5 Gy in 10 to 15 daily fractions, 5 fractions per week. After completing the RT course, patients were followed at 1-2-month intervals with regular brain image surveys until their death. Patients who were unable to present at the out-patient clinic were contacted by telephone.

### 2.4. Evaluation of the Prognostic Factors and Statistics

The clinical factors of the patients were categorized in accordance with previously published articles [3–6]. In addition, the HRV indices including the SDNN and RMSSD were included for survival analysis. The overall survival (OS) was analyzed using the Kaplan-Meier method, and the log-rank test was used to evaluate statistical differences. A multivariate analysis was conducted for all factors by using the Cox's proportional hazard regression method. A significant difference was defined as *P* < 0.05. The analyses were all performed using the SPSS Statistics, version 17.0 (SPSS, Chicago, IL).

## 3. Results

### 3.1. Demographic Data and ECG Analysis

After the first phase of recruitment, the study comprised 51 patients. We excluded 11 patients (21.6%) with arrhythmias or with >1% premature ventricular contractions; thus, 40 patients were included in the final study.

The median age of our study participants was 61 years (range: 39–75). The most common primary cancers among these participants were nonsmall cell carcinoma originating from the lung (24/40) followed by small cell lung cancer (6/40) and breast cancer (4/40). Sixteen patients possessed confirmed brain metastases confirmed at the time of their primary cancer diagnosis. Most of the patients presented extracranial metastases, and their primary sites were not controlled. Only 1 patient was classified as RPA class I (Table 1).

### 3.2. HRV Analysis

Table 2 lists the results of the time domain HRV analysis. Because the NN50 was 0 in 28 patients, the NN50 and pNN50 indices were excluded from the analysis. An SDNN <10 ms or ≥10 ms and RMSSD <7 ms or ≥7 ms were used as prognostic factors in the survival analysis.

### 3.3. Overall Survival and Prognostic Factors

**Figure 1:**
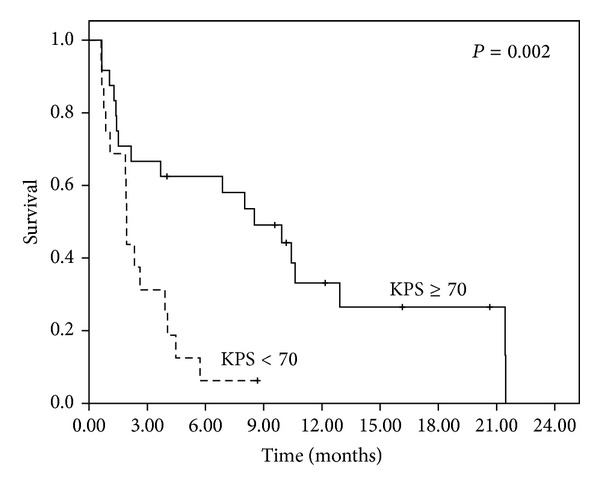
Overall survival of all patients stratified by KPS <70 or KPS ≥70.

**Figure 2:**
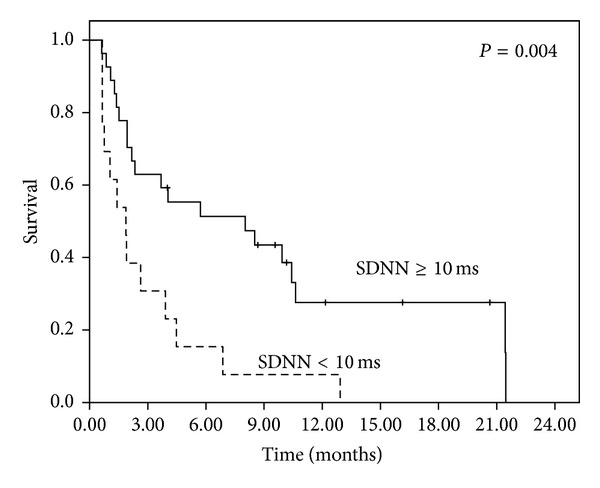
Overall survival of all patients stratified by SDNN <10 ms or SDNN ≥10 ms.

The status of each patient was tracked until death or until the end of the study. At the end of the final analysis in March 2013, 7 patients remained alive. The median follow-up time for all patients was 3.80 months, and it was 10.16 months for the patients who remained alive. In the univariate analysis, only the KPS <70 (*P* = 0.002; Figure 1) and SDNN <10 ms (*P* = 0.004; Figure 2) showed significance for OS (Table 3). The multivariate analysis further confirmed that the KPS <70 (*P* = 0.022) and SDNN <10 ms (*P* = 0.039) were independent prognosticators of OS, exhibiting hazard ratios of 2.657 (95% CI: 1.153–6.123) and 2.204 (95% CI: 1.046–4.733), respectively (Table 3).

## 4. Discussion

The results of this prospective study demonstrate that the HRV index, comprising an SDNN cutoff value of 10 ms, is a novel prognosticator for BM patients, and is independent from the commonly used prognostic indices. To the best of our knowledge, HRV has not been previously documented as a survival prognosticator for BM patients. Because HRV assessment is standardized [10], simple, noninvasive, and cost effective, the potential of this physiological measurement for daily use warrants further investigation.

Among the participants, the median SDNN and RMSSD were 15.0 ms and 10.5 ms, respectively. These values are considerably lower than the published HRV SDNN data from health populations in previous studies [20, 21]. De Couck and Gidron published a large series HRV evaluation of cancer patients (*N* = 657) [15]. After analyzing 10-second ECG recordings, the average patient SDNN value was 21.65 ms, and the values for stage 3-4 patients were significantly lower than those of stage 1-2 patients [15]. In addition, Kim et al. [16] evaluated HRV in 68 terminal cancer patients who were referred for hospice care. The median SDNN and RMSSD were 14.40 ms and 11.35 ms, respectively [16]. The findings in the current study are compatible to these findings, as the patients demonstrated terminal statuses; extremely low HRV values were expected.

However, the mechanism that directly causes the attenuated HRV in BM patients remains unclear. Two possible rationales might explain the relationship: increased intracranial pressure (IICP) and the “vagal-cancer” relationship. The data in previously published studies of brain injury patients have demonstrated a significant correlation between intracranial pressure (ICP) and HRV [22, 23]. In addition to ICP monitor, the authors in Winchell and Hoyt found that patients with IICP had significant attenuated HRV indices, and low HRV correlates with a poor outcome [23]. Biswas et al. reported the same findings in pediatric brain injury patients [22]. The current study did not involve evaluating ICP by the ICP monitor; however, IICP caused by intracranial masses could be expected because the BM patients had never undergone surgical resection of their metastatic tumors. This could explain their attenuated HRV.

The HRV reflects the dynamics of the complex physiological system [10]. It has been demonstrated that vagus nerve activity is highly correlated with HRV [24, 25]. Previous mouse model studies have demonstrated that induced inflammation can cause attenuated HRV through the vagus nerve [26]. It has also been reported that increased oxidative stress is related to decreased HRV [27]. Furthermore, inflammatory reactions and excessive oxidative stress can predispose cancer microenvironments and are related to impaired vagus nerve activity [28]. Based on these previous studies, it is reasonable to doubt that in patients who exhibit a disseminated cancer status and a global physiological environment altered by the cancer, HRV could be attenuated as a consequence of impaired vagus nerve activity. This “vagal-cancer” relationship may explain the low HRV levels in BM patients.

In this study, several time domain HRV indices, such as the TINN, NN50, and pNN50, were not employed for survival analysis. The TINN is an unreliable index for the current study, because a 5-minute ECG recording was not sufficiently long to evaluate this index [10]. The NN50 and pNN50 were not evaluated because 28 patients lacked NN intervals larger than 50 ms (NN50 = 0 and pNN50 = 0).

There are several limitations in this preliminary report. First, the study population was heterogeneous, and we cannot conclude how SDNN would perform in different patient subgroups. Additional studies focusing on these differences are required. Second, the metastatic location was not considered. Although previous study has indicated that intracranial metastatic location is not a survival prognosticator [3] the relationship between intracranial location and HRV remains unknown. We were unable to perform additional analysis because 31 of the participants presented both supratentorial and infratentorial lesions; this issue should be investigated in future studies. Third, the sample size was not sufficiently large and was relatively small compared to previously published studies on BM prognosis [3–6]. The limited number of cases may explain why several documented prognosticators, such as controlled primary, extracranial metastasis, and age, did not exhibit significance in terms of survival. To validate our findings regarding HRV and BM prognosis, additional large-scale studies are warranted. Finally, the HRV analysis is limited to patients who present arrhythmias and ectopic beats, and 21.6% of the patients were not analyzed for HRV. Therefore, alternative HRV analysis tools should be sought for these patients.

In conclusion, the association of HRV as a survival prognosticator in BM patients is studied. The results suggested that SDNN <10 ms may be an independent negative prognosticator of survival. Additional large-scale studies are warranted to evaluate the clinical application of SDNN in the risk stratification of BM patients and as a possible guide for selecting treatment options.

## Figures and Tables

**Table 1:** Baseline patient characteristics (*n* = 40).

Characteristics	No.	(%)
Gender		
Female	21	52.5%
Male	19	47.5%
Age		
Median (range)	61 (39–75)	
<65	24	60.0%
≥65	16	40.0%
Primary		
NSCLC	24	60.0%
SCLC	6	15.0%
Breast cancer	4	10.0%
Others	6	15.0%
BM at diagnosis		
Not	23	57.5%
Yes	17	42.5%
KPS		
Median (range)	70 (30–80)	
<70	16	40.0%
≥70	24	60.0%
Extracranial metastasis		
Without	7	17.5%
With	33	82.5%
Primary status		
Not controlled	35	87.5%
Controlled	5	12.5%
RPA Class		
I	1	2.5%
II	23	57.5%
III	16	40%

NSCLC: Nonsmall cell lung cancer, SCLC: small cell lung cancer, BM at diagnosis: brain metastases confirmed at primary diagnosis, KPS: Karnofsky performance status, and RPA: recursive partitioning analysis.

**Table 2:** Time domain HRV analysis (*n* = 40).

HRV Indices	Mean	SD	Median	Lower one-third	Upper one-third
Mean NN (ms)	733.58	136.04	712.50	669.00	788.54
SDNN (ms)	14.30	7.68	15.00	10.06	17.06
RMSSD (ms)	11.65	7.88	10.50	6.53	15.06
TINN (ms)	76.65	28.41	78.00	63.00	94.00
NN50	2.45	5.34	0.00	0.00	0.00
pNN50 (%)	0.63	1.33	0.00	0.00	0.00

HRV: heart rate variability, SD: standard deviation, NN: normal-to-normal interval, SDNN: standard deviation of normal-to-normal interval, RMSSD: root mean square standard deviation of normal-to-normal interval, TINN: triangular interpolation of normal-to-normal interval histogram, NN50: number of pairs of adjacent normal-to-normal intervals differing by more than 50 ms, and pNN50: the proportion of NN50 divided by total normal-to-normal intervals.

**Table 3:** Prognostic factors for overall survival-univariate analysis and multivariate analysis.

Characteristics	Univariate analysis				Multivariate analysis	
	3M survival (%)	6M survival (%)	Median survival (M)	*P* value	HR (95% CI)	*P* value
Gender				0.711		0.604
Female	57.1	42.9	3.9		(—)	
Male	47.4	35.5	2.0			
Age				0.283		0.303
<65	54.2	45.8	3.9		(—)	
≥65	50.0	29.2	2.6			
Primary				0.160		0.333
NSCLC	54.2	48.5	3.7		(—)	
Others	50.0	29.2	1.9			
BM at diagnosis				0.996		0.775
No	50.0	36.7	2.3		(—)	
Yes	56.3	37.5	3.7			
KPS				0.002		0.022
<70	31.3	0.63	1.9		2.657 (1.153–6.123)	
≥70	66.7	62.5	8.5		Reference	
Extracranial metastasis				0.087		0.171
Without	71.4	57.1	21.4		(—)	
With	48.5	36.4	2.6			
Primary status				0.400		0.097
Not controlled	51.4	39.5	3.7		(—)	
Controlled	60.0	40.0	3.9			
SDNN (ms)				0.004		0.039
<10	30.8	15.4	1.8		2.204 (1.046–4.733)	
≥10	63.0	51.4	8.0		Reference	
RMSSD (ms)				0.100		0.868
<7	42.9	21.4	1.9		(—)	
≥7	57.7	49.7	5.7			

HR: hazard ratio, CI: confidence interval, NSCLC: Nonsmall cell lung cancer, BM at Diagnosis: brain metastases confirmed at primary diagnosis, KPS: Karnofsky performance status, SDNN: standard deviation of normal-to-normal interval, and RMSSD: root mean square standard deviation of normal-to-normal interval.
